# Responses of Red Flour Beetle Adults, *Tribolium castaneum* (Coleoptera: Tenebrionidae), and Other Stored Product Beetles to Different Pheromone Trap Designs

**DOI:** 10.3390/insects11110733

**Published:** 2020-10-27

**Authors:** Carl W. Doud, Thomas W. Phillips

**Affiliations:** 1 Department of Entomology, 123 Waters Hall, Kansas State University, Manhattan, KS 66506, USA; cdoud@co.midland.mi.us; 2 Mosquito Control Midland County, 2180 N Meridian Road, Sanford, MI 48657, USA

**Keywords:** *Typhaea stercorea*, *Ahasverus advena*, food baits, funnel trap, flourmill, stored products

## Abstract

**Simple Summary:**

Traps are used to monitor insect pests in stored food product habitats, and information on insects trapped can be used for making control decisions in pest control programs. A special trap is used for adults of the red flour beetle, one of the most serious pests of flourmills worldwide. The red flour beetle trap is a “pitfall” design wherein a walking beetle orients to the trap, it climbs up the inclined side of the trap to the top of a cup, it then slips or walks over the edge of the cup. The beetle falls into the bottom of the cup, where it is retained and killed inside the food oil placed in the bottom of the cup. However, dusty environments can result in the pitfall trap operating poorly and not capturing beetles at an optimum level. This research showed that a dust cover can be applied to the top of a baited pitfall trap and significantly improve capture of beetles in dusty environments of a flourmill. The dust cover was incorporated into the commercial pitfall trap product and now is known as the Dome trap, which is widely used for pest management of flour beetles and other stored product insects throughout the grain and food industries in many countries.

**Abstract:**

A series of laboratory and field experiments were performed to assess the responses of *Tribolium castaneum* (Herbst) and other stored-product beetles to pheromone-baited traps and trap components. A commercial *Tribolium* pitfall trap called the Flit-Trak M^2^, the predecessor to the Dome trap, was superior in both laboratory and field experiments over the other floor trap designs assessed at capturing walking *T. castaneum*. In field experiments, *Typhaea stercorea* (L.) and *Ahasverus advena* (Stephens) both preferred a sticky trap to the pitfall trap. Although the covered trap is effective at capturing several other species of stored product beetles, the synthetic *Tribolium* aggregation pheromone lure is critical for the pitfall trap’s efficacy for *T. castaneum*. Although the food-based trapping oil used in the pitfall trap was not found to be attractive to *T. castaneum* when assayed alone, it had value as an enhancer of the pheromone bait when the two were used together in the trap. A dust cover modification made to go over the pitfall trap was effective in protecting the trap from dust, although the trap was still vulnerable to dust contamination from sanitation techniques that used compressed air to blow down the mill floors. Capture of *T. castaneum* in the modified trap performed as well as the standard trap design in a non-dusty area of a flour mill, and was significantly superior over the standard trap in a dusty area. *T. castaneum* responded in flight outside a flourmill preferentially to multiple funnel traps with pheromone lures compared to traps without pheromone.

## 1. Introduction

Beetles of the genus *Tribolium*, in particular the red flour beetle, *Tribolium castaneum* (Herbst) and the confused flour beetle, *Tribolium confusum* Jacquelin du Val, (Coleoptera: Tenebrionidae), are major pests of many durable post-harvest food commodities. A current method of monitoring *Tribolium* spp. is the use of pheromone and food-oil baited traps [1,2,3]. Males of *T. castaneum* produce an aggregation pheromone, 4,8-dimethyldecanal (DMD), when actively feeding at relatively low density [4,5]. Suzuki [6] first identified and synthesized this pheromone as 4,8-dimethyldecanal (DMD) from males of both *T. castaneum* and *T. confusum*.

One of the most commonly used *Tribolium* traps in the USA and other countries is the so-called Dome trap [7]. The initial commercial version and predecessor to the Dome trap was the Storgard Flit Trak^®^ M^2^ (Trécé, Inc. Salinas, CA, USA; now located in Adair, OK, USA), and it was based on the prototype designed by Mullen [8]. The trap is a ramp-pitfall design that utilizes both DMD pheromone being released from an impregnated rubber septum, and a food-based oil as attractants [9,10,11]. The exact composition of the food oil that is sold with the trap is proprietary information, but we were told it contains a grain-based food oil, such as wheat germ oil (Bill Lindgren, personal communication). Other common trap designs include floor-mounted adhesive traps that are used either unbaited or baited with *Tribolium* pheromone and food odor lures [11,12].

The trapping environment can affect the efficacy and useful longevity of traps (Barak et al., 1991). Tingle and Mitchell [13] demonstrated during trapping studies with moths that adhesive sticky traps could be detrimentally affected by dust. Large amounts of flour dust are produced as a result of the milling processes in flourmills and has been shown to lower the efficacy of sticky and pitfall traps [14]. These observations led to the development of a dust-cover modification to the pitfall trap, which is described in the research below.

Our overall objective was to determine if traps of different designs for stored product beetles are effective for capturing adult *T. castaneum*, and also to determine if one commonly used trap could be improved with simple design modifications. The specific objectives of this research were: (a) compare the efficacy of sticky traps and pitfall traps for the capture of walking *T. castaneum*; (b) determine if the food oil used for pitfall traps contributes to the attractiveness of the synthetic aggregation pheromones to *T. castaneum*; (c) assess the benefit of using a dust cover on a pitfall trap to improve the trapping efficacy of *T. castaneum* in the field; and (d) determine if *T. castaneum* could be captured in flight outdoors using the aggregation pheromone with flight traps. Much of this work was reported in the M.S. thesis of Doud [14] and is now presented here in final form for peer-reviewed publication.

## 2. Materials and Methods

### 2.1. Traps and Pheromone Lures

**Figure 1:**
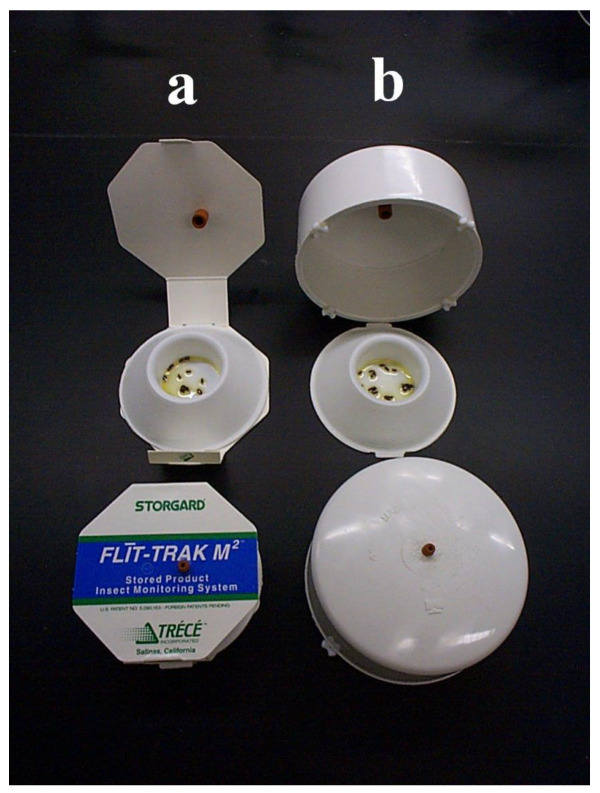
Designs of pitfall traps studied. (**a**) The standard pitfall trap was the Flit-Trak M^2^ from Trecé Inc. with a cardboard cover. (**b**) The modified pitfall trap used the same ramp-pitfall component but with the cardboard cover replaced with a solid cover made from a polyvinyl chloride (PVC) pipe end-cap and four plastic beads as supports to raise the cover 3 mm above the floor.

Experiments were conducted with a standard and modified pitfall trap, three different sticky traps, and a multiple funnel trap for flying insects. The pitfall trap was the Flit-Trak M^2^ from Trécé Inc. and consisted of a plastic cup with the side-wall ramped from the diameter at the base of 9.5 cm and decreasing to the top of the cup with a 5.0 cm diameter. The surface of the ramp was rough to allow insects to crawl up the ramp. The top of the ramp and inside wall of the cup had a smooth surface so that responding beetles would fall into the cup and onto the cup’s floor, and be unable to crawl up and out of the cup (Figure 1a). Approximately 0.5 mL of the food/oil bait provided by the manufacturer was placed into the floor of the pitfall cup to trap and kill responding beetles. The standard pitfall trap came with a cardboard cover that suspended the pheromone lure over the cup and provided some protection from dust and debris, a supply of oil, and a lure that was a rubber injection septum impregnated with the synthetic pheromone, 4, 8 dimethyldecanal in a blend of optical isomers at 4RR and 8RS, referred to commonly with the acronym DMD. The modified pitfall trap had a 10 cm inside diameter polyvinyl chloride pipe end-cap to replace the standard cardboard cover (Figure 1b). The cap rested on four plastic beads glued on its lower rim to allow for a 3-mm clearance for beetles to contact the ramp-pitfall cup protected under the cap. The top of the cap had a small hole in the center to hold the pheromone lure.

**Figure 2:**
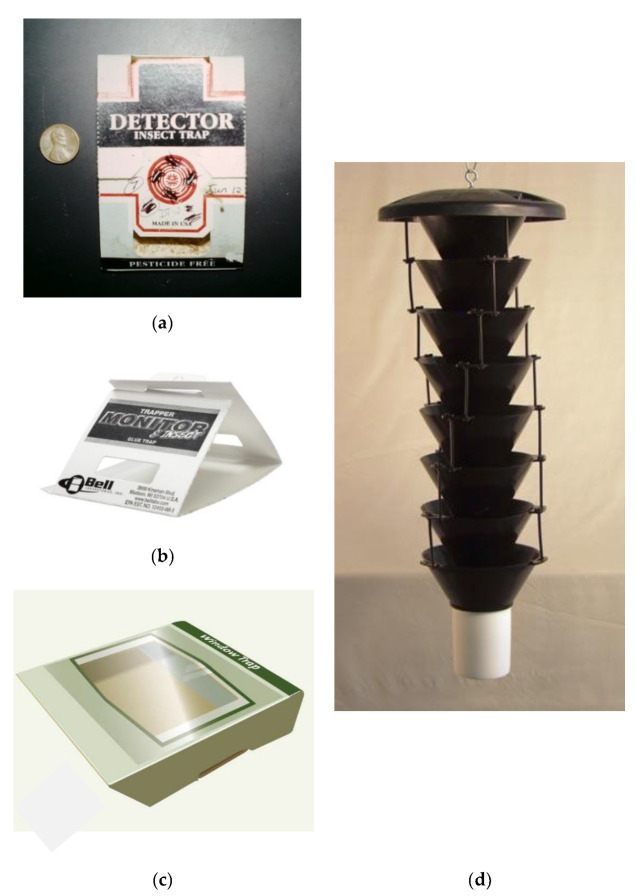
Sticky traps and a flight trap studied. Detector Trap (**a**), approximately 9.5 × 6.5 cm with a height of 1.0 cm max. Walking insects enter from right or left and are caught on the sticky floor. Trapper Monitor (**b**), approximately 8.0 × 8.0 cm at bottom and up to 6.0 cm high when set up. All three inside surfaces have sticky material. The Window trap (**c**), approximately 8.0 × 8.0 cm with a height of approximately 1.0 cm; inside bottom is sticky. Insects enter the sides, walk up a small ramp and then walk or fall onto the sticky floor. Lindgren multiple funnel trap (**d**) with plastic funnels, each funnel approximately 20 cm diameter at top and 10 cm at the bottom opening. Pictured is an 8-unit trap as used in this study. The white plastic collection cup is bayonet-mounted to the bottom trap.

Three different sticky traps were evaluated in these experiments (Figure 2). The Detector trap (AgriSense, Palo Alto, CA, USA) was made of cardboard with a 7.5 × 6.5 cm inside sticky floor surface. The top of the Detector trap was folded in on itself to connect with the trap bottom, provide some protection from debris to the sticky surface, and allow entry of walking beetles on the two sides. The Trapper Monitor insect trap was provided by Bell Laboratories (Madison, WI, USA). This trap was also made of cardboard with a 7.5 × 7.5 cm inside sticky floor surface and entry of walking beetles was provided on the two sides. AgriSense also provided the Window Sticky Trap, which also had a floor surface of 7.5 × 7.5 cm. The trap’s sticky surface was enclosed under a transparent plastic cover approximately 6 mm above the floor of the trap. Beetles entered the trap through short ramps positioned on the two open sides of the trap, and then stepped or fell off the ramp onto the sticky surface.

Multiple funnel traps were originally developed for capturing flying bark and ambrosia beetles (Coleoptera: Curculionidae: Scolytinae) [15] and were purchased from Phero-Tech Inc. (now Contech, Inc., Delta, BC, Canada). The funnel traps had eight black plastic funnels approximately 24 cm diameter at the top and with a 10 cm diameter hole at the bottom (Figure 2d).

Insects flying into the trap land on or strike the outside of any of the top seven funnels and then fall through the lower funnels into the collecting cup, 10 cm dia × 12 cm tall, at the bottom of the trap. Small pesticide strips releasing vapors of dichlorvos (e.g., a product similar to http://www.hotshot.com/products/general-insect-control/no-pest-strip.aspx) insecticide were placed in the cup to kill captured insects.

### 2.2. Laboratory Bioassays

All laboratory experiments were conducted using an established laboratory colony of *T. castaneum* reared on whole-wheat flour and brewer’s yeast (95:5) in standard glass canning jars (0.95 L). Colonies were maintained in a growth chamber at 28 °C, 70% relative humidity, in complete darkness. Adult beetles used in bioassays were four to six weeks old and were separated without food from their colonies 24 h prior to the experiment to enhance their olfactory response, and to ensure uniform starvation.

A metal tray was used as an orientation arena for bioassays of *T. castaneum* orientation to different trap designs and trap components [4]. Each experiment used several stainless-steel trays, each 92 × 92 × 9 cm with one layer of whole-wheat grain kernels covering the floor. The layer of grain was included to simulate a natural stored-grain environment for the responding beetles and to provide a surface conducive for walking by adult *T. castaneum*. Each tray was an experimental unit with one trap placed on the floor of the tray at a randomly selected location that was at least 15 cm from one of the four sides of the tray. Each tray was deployed with just one trap to evaluate beetle orientation, thus the design was a “no-choice” with regard to what the beetles could orient to. Laboratory experiments were set up in a randomized complete block design with blocks set up on separate days. Subsequent complete blocks of trays were randomized for trap placement in each tray and location of trays with different treatments placed at randomly selected locations in our bioassay room. Approximately 100 mixed-sex adult *T. castaneum* were put in the center of a tray under an inverted 6 cm plastic Petri dish for 15 min once the trays and traps were prepared for testing on a table in the isolated bioassay room. The bioassay began after the 15-min acclimation period when beetles were released from under the dish and a screen cover was then placed over the tray to prevent escape of beetles. A block of tray bioassays was conducted for 20 h, from 1400 h on the first day to 1000 h of the following day, in a room with total darkness at 28 ± 2 °C and 70 ± 10% r.h. The number of beetles captured in each trap was recorded and the beetles remaining in the tray were sifted from the wheat and counted. The proportion of beetles captured relative to the total number of beetles released in the tray was computed. Wheat and beetles for each tray assay were used only once, the plastic release dish was discarded, and the trays were thoroughly washed and dried before re-use.

A series of four experiments were conducted using the metal-tray bioassay. In the first experiment, the responses of *T. castaneum* to trap designs without any pheromone or food oils were assessed. The treatments were: (1) Detector sticky trap unbaited, (2) Trapper Monitor sticky trap unbaited, and (3) pitfall trap unbaited (mineral oil was used in the cup reservoir as a non-food-derived trapping medium). The experiment was replicated four times. A second experiment compared the response of the beetles to the Trapper and Window sticky traps, with and without a pheromone lure placed in the center of the sticky surface. The treatments were: (1) Trapper sticky trap without pheromone, (2) Trapper sticky trap with a pheromone lure, (3) Window trap without pheromone, and (4) Window trap with a pheromone lure. The experiment was replicated four times. In the third laboratory experiment, the responses of *T. castaneum* to each of the pitfall trap semiochemical components was assessed. The standard pitfall trap Flit-Trak M^2^ with the cardboard cover was used. Treatments compared were: (1) a control trap with only 0.5 mL mineral oil to retain and kill the trapped beetles, (2) a trap with 0.5 mL of the commercially provided food oil bait only and no pheromone lure, (3) a trap with pheromone only with mineral oil in place of food oil, and (4) a trap with both the pheromone lure and food oil. The experiment was replicated eight times. The fourth laboratory experiment compared the standard pitfall trap with the cardboard cover, Flit-Trak M^2^, to the modified trap equipped with the PVC cap. Both trap treatments used the standard pheromone and food oil lures. The experiment was replicated eight times.

We analyzed results from each of the laboratory tray experiments using SAS [16] by an analysis of variance (ANOVA) using PROC MIXED on arcsine square root transformed proportion for counts of beetles in a trap relative to total beetles in the tray. Significant ANOVA results were then subjected to a least significant difference (LSD) separation test for experiments with more than two treatments.

### 2.3. Field Experiments

Four separate experiments were done at a commercial wheat flourmill. The manager of the mill reported to us that the mill produced 750,000 kg of flour per day and operated 24 h a day, seven days a week during the time of this research [14]. Traps were deployed throughout the eight floors of the mill, which had the facilities for processing wheat into flour and storage of bulk and packaged finished commodities. Floor areas in the mill were approximately 25 × 50 m in size. Thirty-six bins were attached to the main building for storing and distributing newly made flour over a 7–14-day period. Average high and low temperatures and humidity inside the mill were recorded by us with HOBO^®^ data loggers (Onset Computer Corporation, Bourne, MA, USA) and these values ranged from 20–42 °C and 15–70% RH, while conditions during the one outdoor experiment ranged from 22–40 °C in July to 11–22 °C in October with RH values between 10% and 55%. The mill’s manager reported *T. castaneum* as their most important pest but that other stored product pests were present.

The first field experiment compared the standard Flit-Trak M^2^ pitfall trap to the Detector sticky trap for responses of *T. castaneum*. *Typhaea stercorea* (L.) (Coleoptera: Mycetophagidae) and *Ahasverus advena* (Stephens) (Coleoptera: Silvanidae) were unexpectedly captured in this experiment so their responses were also evaluated. Treatment blocks had the two traps spaced at 3 m between them and placed along a wall, with 6 m or more between blocks, and their locations were randomly selected on five mill processing floors, as well as in an area above the bulk stored flour bins. Trap block locations were not changed during the study. A total of 26 of the 2-trap blocks were placed throughout the mill, trapped insects were removed and counted every two weeks, the position of each trap in a pair was randomly selected at the time of servicing every two weeks, and pheromone lures were replaced every four weeks. The contents of the pitfall traps were placed in a ziplock bag and taken back to the laboratory for identification while the sticky floor traps were collected and replaced with a new trap at each 2-week check time. Numbers of beetles trapped in this experiment were very low, so the means of beetles per trap were square root transformed before subjecting to ANOVA (PROC MIXED) for differences between traps in a pair.

The second experiment aimed to quantify and compare the amount of dust accumulated in the pitfall cups of the standard trap that had the opened cardboard cover (Figure 1, left) and the modified pitfall trap with the domed PVC end-cap cover (Figure 1, right) in four locations of the mill over a 7-day period. The two traps were randomly placed in each of seven blocks (replicates) for each of the four mill locations. Each two-trap block in all four mill locations had 3 m between the two traps in a block, and at least 6 m spacing between blocks. Mineral oil rather than food oil (0.5 mL) was placed in the collection cups of both trap types. Four distinct areas of the mill were chosen to place the traps based on perceived variation in dustiness: (1) the third floor of the mill, which was very dusty and cleaned daily with a compressed air blow-down; (2) the second floor packing area, which was also dusty and was cleaned once a week with compressed air blow-down; and (3) the small room at the bottom of the bulk-stored flour bins, which was extremely dusty from hopper unloading and pneumatic transfer of flour. This particular area was not cleaned during this experiment. The fourth trapping area was the finished product warehouse area that was relatively dust-free. Each pitfall cup was weighed in a sealed plastic sandwich bags prior to deployment in the mill, and again after collecting and its return to the laboratory. Any insects trapped during the study were removed to account only for increased weight of the trap due to accumulated dust and debris. Differences in weight of accumulated flour in standard vs. covered pitfall traps were analyzed using ANOVA for each of the trapping locations.

The responses of *T. castaneum* to the standard Flit-Trak M^2^ pitfall design vs. the modified pitfall traps were evaluated in a third experiment at the low-dust finished product warehouse (12 replicates) and in the high-dust “feed warehouse” (4 replicates), which was a room holding bran and damaged grains destined for feed mills. Differences in numbers of*T. castaneum* caught in standard vs. covered pitfall traps were analyzed using ANOVA for each of the two trapping locations.

A fourth indoor experiment used a two-trap comparison to study the role of the pheromone lure in captures of *T. castaneum* using modified pitfall traps at three locations in the flourmill that differed in dustiness. Treatments were simply a trap with a pheromone lure and food-oil in the cup, and a trap without the pheromone lure but also with the food oil. Traps within a pair were 3 m apart and there was 6 m or more between pairs of traps. We deployed six replicates in each of the three areas. Differences in *T. castaneum* beetles caught in pheromone-baited traps vs. traps not baited with pheromone were analyzed using ANOVA for each of the trapping locations.

The flight response of *T. castaneum* to the eight-unit funnel traps was assessed in an outdoor experiment approximately 100 m north of the flourmill. Treatments were deployed in a randomized complete block design consisting of a trap with no pheromone and one with a standard pheromone lure (as used for pitfall traps) that was hung by a wire from the bottom funnel. Traps were placed ~2 m above the ground attached to either a chain-link fence at the edge of the flourmill property or on utility poles within the mill yard. The two traps in a block were spaced approximately 10 m apart, blocks were spaced at least 15 m apart and monitored for the months of June, July, August, September, and October in 1998. Traps for blocks one and two were placed on 16 June 1998. Traps were checked and re-randomized weekly within each block, and trapped insects were taken to the lab for identification. Data were sorted and analyzed by month for four separate analyses. Differences in *T. castaneum* beetles caught in pheromone-baited traps vs. traps not baited with pheromone were analyzed for each month using ANOVA.

## 3. Results

### 3.1. Response of T. castaneum to Traps in Laboratory Assays

**Figure 3:**
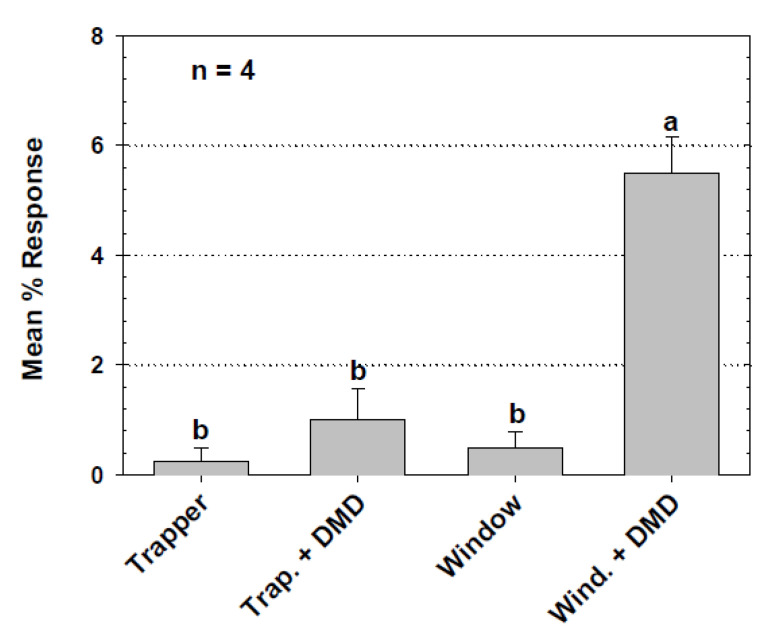
Mean (SE)% responses of *T. castaneum* to Trapper and Window sticky traps, with and without DMD lures, in the metal tray laboratory bioassay. Percent data are based on the proportion of the number of beetles in the trap after 20 h relative to the total number released into the tray. ANOVA revealed a significant pheromone by trap interaction (*F* = 20.16; df = 1,9; *p* = 0.0015). Means with the same letter are not significantly different (*p* > 0.05). Percent captures were arcsine square root transformed for ANOVA but means of untransformed numbers are reported in the figure.

**Figure 4:**
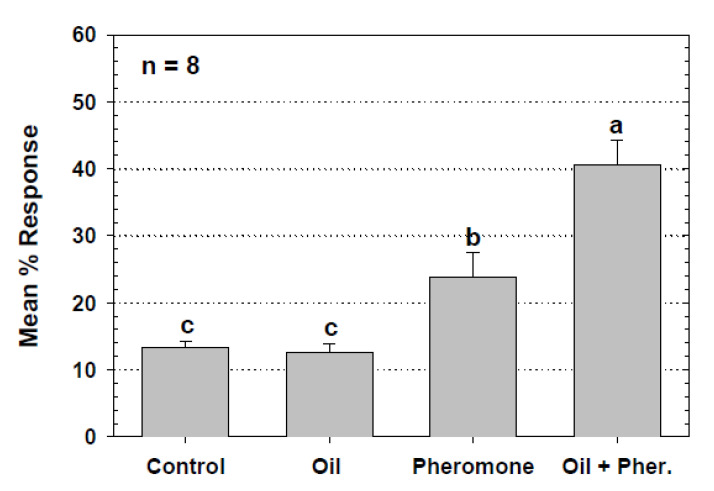
Mean (SE)% responses of *T. castaneum* to the semiochemicals provided with the commercial pitfall trap in the metal tray laboratory bioassay. Treatments were: (1) control (1 mL mineral oil only), (2) Oil (1 mL trapping oil, no pheromone lure), (3) Pheromone (1 mL mineral oil, pheromone lure), and (4) Oil + Pher. (1 mL Storgard trapping oil, pheromone lure). Percent data are based on the number of beetles trapped after 20 h relative to the total number released into the tray. ANOVA revealed a significant interaction between oil and pheromone in the trap (*F* = 12.77; df = 1,21; *p* = 0.0018). Means with the same letter are not significantly different (*p* > 0.05). Percent captures were arcsine square root transformed for ANOVA but means of untransformed numbers are reported in the figure.

The results of the experiment to assess the response of *T. castaneum* to the pitfall trap and two sticky traps without pheromone or other attractants, revealed a significant difference in capture (*F* = 18.84; df = 2,3; *p* = 0.003) among the three trap types. Significantly more beetles were captured by the pitfall trap with an average of 21.25 (±4.13 SE)% captured in each tray that had a pitfall trap. The Detector sticky trap caught 5.00 (±0.91 SE)% of released beetles and was not significantly different from the Trapper Monitor sticky trap, which caught 1.50 (±0.29 SE)%, while the proportions trapped in both sticky traps were significantly less than those caught in the pitfall trap (LSD test, *p* < 0.05). The response of *T. castaneum* to the Trapper and the Window sticky traps, with and without pheromone lures, was very low for all treatments. However, the Window sticky trap caught significantly more beetles with pheromone than without, while the Trapper sticky trap caught low numbers of beetles whether deployed with a pheromone lure or without (Figure 3). The experiment comparing captures in the standard trap with or without attractants found that that traps with food oil only captured small numbers of *T. castaneum* that did not differ statistically from numbers caught in unbaited control traps with mineral oil (Figure 4). Traps with pheromone alone captured significantly more beetles than the control, and traps baited with both food oil and a pheromone lure caught significantly more beetles than other treatments. When the standard pitfall trap with the cardboard cover was compared to the modified pitfall trap with the PVC cover in the metal tray laboratory assay, each with food oil and a pheromone lure, the two had statistically similar capture of *T. castaneum*. The standard cardboard-covered trap captured a mean of 23.09 (±5.10 SE)% of released beetles and the modified trap captured 26.09 (±3.51 SE)% of released beetles (*F* = 2.08 df = 1, 7; *p* = 0.217).

### 3.2. Trapping Experiments at a Flourmill

**Figure 5:**
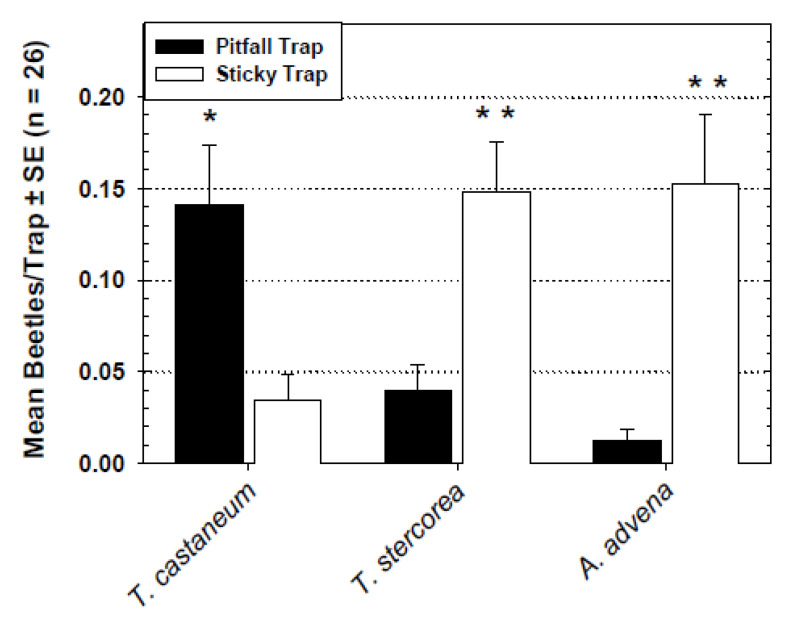
Captures of three species of stored-product beetles by standard pitfall and Detector sticky trap designs in the flourmill; trap-catch data analyzed separately for each species. Significantly more beetles were captured in the pitfall traps compared to the sticky traps for *T. castaneum* (*F* = 6.59; df = 1, 25; * = *p* < 0.05), the sticky traps over pitfall traps for *T. stercorea* (*F* = 10.92; df = 1, 25; ** = *p* < 0.01), and sticky traps over pitfall traps for *A. advena* (*F* = 10.95; df = 1, 25; ** = *p* < 0.01).

The capture of *T. castaneum*, *T. stercorea*, and *A. advena* by the Detector sticky floor trap and the original pitfall trap within a flourmill are plotted in Figure 5. Captures of *T. castaneum* by the pitfall trap were significantly higher than captures in the Detector sticky trap. However, *T. stercorea* and *A. advena* were captured in significantly higher numbers in the Detector sticky trap compared to the pitfall trap.

**Figure 6:**
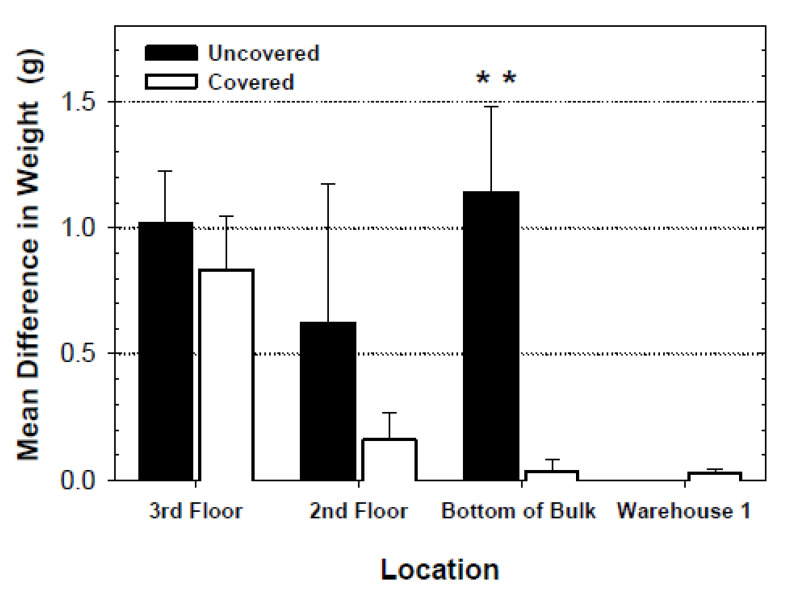
Increase in mean (+/−SE) weight (g), due to the accumulation of flour dust and debris, of uncovered and covered pitfall traps after one week on the floor at four locations in the flourmill known to vary in dustiness. The analyses of trap weight comparisons were made as separate paired comparisons for each of the four locations. Uncovered traps weighed significantly more than covered traps in the area below the bulk flour bins (*F* = 18.59; df = 1, 6; ** = *p* < 0.01). Differences of trap weights in other locations were not statistically significant.

**Figure 7:**
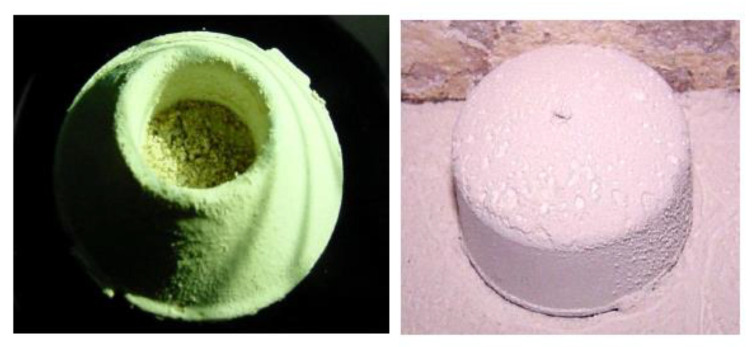
Two types of traps exposed in the flourmill for one week. The pitfall cup from a regular pitfall trap equipped with its standard cardboard cover and placed on the floor in the room under the bulk flour storage is on the left, and a modified trap with the durable plastic cover made from a PVC pipe endcap at right at the same location. The gain of weight reported in Figure 6 for the uncovered pitfall traps in the “Bottom of Bulk” was due to accumulation of flour dust and debris, which was apparently prevented in the traps with the PVC cover.

**Figure 8:**
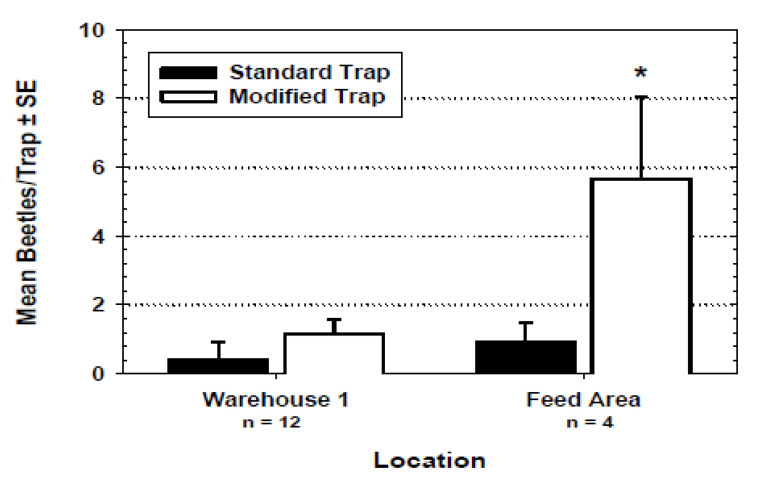
Capture of *T. castaneum* by standard pitfall traps with cardboard covers and modified pitfall traps at two locations in the flourmill. Capture was not significantly different in warehouse 1 (*F* = 2.14; df = 1, 11; *p* = 0.1097). In the feed area, the modified trap captured significantly more beetles (*F* = 4.79; df = 1, 3; * = *p* < 0.05). Count data were square root transformed for ANOVA but mean values of whole numbers are reported here.

Dust accumulation in the standard Flit-Trak M^2^ pitfall trap and the modified trap varied among locations in the flourmill (Figure 6 and Figure 7). No significant difference was observed in the increase of trap weights on the third floor of the mill (*F* = 0.22; df = 1, 5; *p* = 0.6584), and second floor (*F* = 2.18; df = 1, 5; *p* = 0.1907) as well as in the finished product warehouse (*F* = 0.06; df = 1, 5; *p* = 0.8169). Traps placed on the floor below the bulk storage area had a significant difference in weight gain in standard uncovered traps compared to PVC-covered traps. The relative dustiness of each mill location can be observed by noting the uncovered trap weight difference for each of the four mill locations. The dustier areas of the mill (third and second floors and the area below bulk flour bins) experienced an increase in weight due to dust of anywhere from 0.62 to 1.14 g. The average weight change for the uncovered traps in the warehouse area was not different. When the standard and modified traps were compared in the non-dusty warehouse 1 and in the dusty feed area of mill 1, there were significantly more beetles captured by modified traps in the feed area (Figure 8).

**Figure 9:**
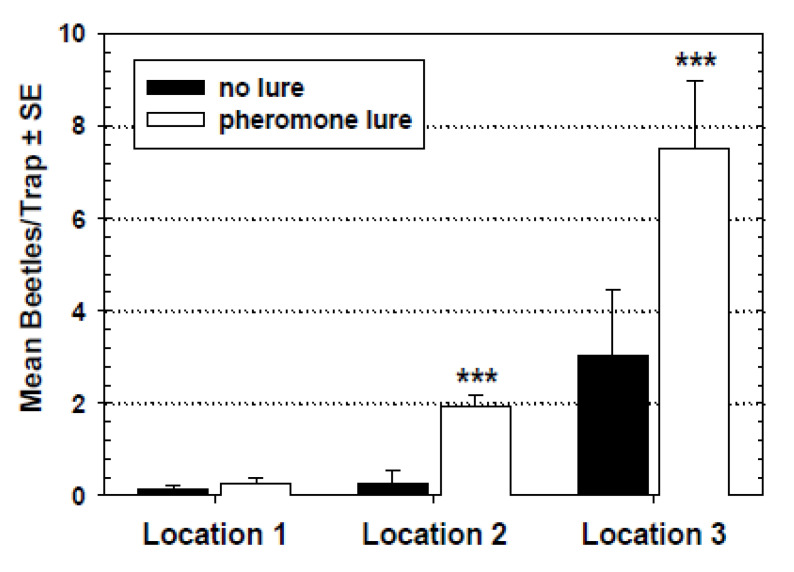
Capture of *T. castaneum* in modified pitfall traps with or without DMD lures within three locations of a flourmill. ANOVA determined that capture was not significantly different in location 1 (*F* = 0.48; df = 1, 5; *p* = 0.52) but was significantly higher in pheromone traps in location 2 (*F* = 115.65; df = 1, 5; *** = *p* < 0.001) and location 3 (*F* = 475.36; df = 1, 5; *** = *p* < 0.001).

When the capture of the *T. castaneum* by modified pitfall traps with oil and pheromone lures was compared to that of the same trap design without the pheromone lure, there was no significant difference in capture between the two traps in location 1 (Figure 9). However, capture was significantly different within location 2 and location 3.

**Figure 10:**
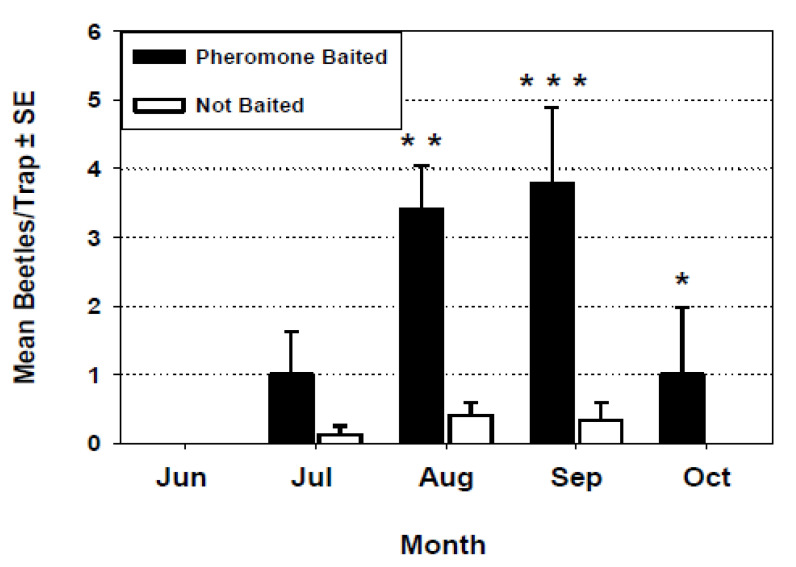
Capture of *T. castaneum* in multiple funnel traps with and without pheromone lures outside the flourmill. Capture was not significantly different among trap treatments during July (*F* = 3.06; df = 1, 3; *p* = 0.1071). Capture was significantly higher in pheromone traps during August (*F* = 18.32; df = 1, 3; ** = *p* < 0.01), September (*F* = 28.56; df = 1, 3; *** = *p* < 0.001), and October (*F* = 5.82; df = 1, 3; * = *p* < 0.05).

Pairs of multiple funnel traps deployed outside the flourmill, either with or without pheromone lures, captured *T. castaneum* in flight. Captures were low and not significantly different between baited and unbaited traps during July (Figure 10) but were significantly higher in the pheromone-baited traps during August, September, and October.

## 4. Discussion

The standard Flit-Trak M^2^ pitfall trap proved superior to the Detector and Trapper sticky traps in capture of *T. castaneum* in all experiments that compared them. This result is similar to what was observed by Mullen [8] during the development of the pitfall trap. Interestingly, our work found that *T. stercorea* and *A. advena* were captured more by the Detector sticky trap over the pitfall trap in the flour mill assessment, suggesting that the behavior of these two beetle species in response to traps is different than that of *T. castaneum*. Perhaps these two beetles are simply more susceptible to the sticky traps and perhaps do not climb to the top of the pitfall cup like *T. castaneum*. Stejskal [17] studied response of *T. castaneum* to sticky traps under highly controlled laboratory conditions, but we are not aware of studies that have looked at responses of stored product beetles in laboratory choice studies and commercial settings like the flourmill work we did here. Addition of synthetic pheromone to the Window sticky trap elicited a larger response by beetles in our lab assays, but addition of pheromone to the Trapper sticky trap did not result in increased activity (Figure 3). Responses of *T. castaneum* were higher to pheromone-baited pitfall traps than to pheromone-baited sticky Detector traps in the flourmill, but responses of *T. stercorea* and *A. advena* were greater to the Detector sticky traps than to pitfall traps. We do not know if *T. stercorea* and *A. advena* respond preferentially to the *Tribolium* pheromone compared to traps with no pheromone, and this possibility could be tested in future work. Subsequent research using the current Dome design of the Trécé pitfall trap revealed that despite having species-specific pheromone lures, many non-target species of beetles and other insects can be trapped in these pitfall traps [18,19]. Therefore, the pitfall trap can serve as a monitoring tool for targeted species and also for other stored product pests that may be present in an area being studied.

Interesting results were observed from experiments to isolate and evaluate specific pitfall trap components. The four treatment assessment of the pitfall trap and its semiochemicals in the laboratory revealed that the trapping oil alone did not attract *T. castaneum*; however, when used in combination with the pheromone lure the oil was able to significantly increase capture over the trap with pheromone alone (Figure 4). The pheromone used in these pitfall traps was a synthetic mimic of the natural male-produced pheromone. Males produce the pheromone only when feeding, and the resulting attractants elicit responses by both males and females, thus its designation as an aggregation pheromone [2,5,7]. Results from the experiment done in the mill to compare capture of *T. castaneum* in modified pitfall traps with and without pheromone lures revealed a significantly higher capture by the traps with pheromone in two of three locations (Figure 9). It is likely that the first location (third floor of mill) did not support a high enough beetle population to reveal differences in trap capture. Another possibility for the lack of significant difference is that the traps in this area may have been lower in efficacy due to dusty conditions and mill floor cleaning techniques (see below).

We did not determine the sex of beetles responding to traps baited with the combination of synthetic pheromone and the food oil, but we predict that the sex ratio may have been female biased. This would be a good topic for future research. The phenomenon of male beetles feeding on host material and then releasing an aggregation pheromone that attracts females is common throughout the Coleotpera that have been studied [2,20,21]. Females need suitable host plant material in which to lay eggs and reproduce. Males that can attract females to such host material may have improved reproductive fitness if they can mate with a responding female before she oviposits. Other males responding to the male-produced pheromone are taking advantage of this signal to have the opportunity to mate females before they lay eggs. Therefore, the adaptive nature of good responses by beetles to a combination of a pheromone with volatiles from a host resource provides information for developing optimal traps to detect and monitor a specific pest for pest management purposes.

The PVC end-cap modification to the standard pitfall trap reduced the amount of flour dust accumulating on the trap and it generally improved trap efficacy in the dusty mill environments. The modified trap showed no inhibitory effects in the capture of *T. castaneum* in dust-free laboratory metal trays nor in any of the field environments. Furthermore, when these two traps were compared in a dusty area of the flourmill, the modified trap captured significantly more beetles (Figure 8). The dust accumulation study produced interesting results regarding various areas of the flourmill, and the efficacy of the dust cover trap modification in protecting the trap was confirmed. The third-floor traps experienced a relatively large amount of weight gain due to dust, apparently from the daily blow-down cleanings, and the dust cover did not offer protection from the dust as evidenced by the statistically similar amount of weight increase of both covered and uncovered traps (Figure 6). However, the area below the bulk flour bins that was not cleaned during our study did have a significantly different weight increase. Noteworthy as well is that some of the uncovered traps in the bottom of the bulk experienced the greatest dust weight increase of all three areas studied, as evidenced by the higher error bar. Therefore, the PVC cover was apparently efficient in protecting the trap from dust. This difference can be attributed to the frequency and technique of floor cleaning used in these areas. The area below the bulk stored flour, although being the area of greatest dust accumulation, was undisturbed by blow-down cleaning during the study. Conversely, we were told by flourmill staff that the third floor of the mill was cleaned daily during the study with compressed air blow-downs. These blow-downs possibly forced dust up under the cap, causing an increase in weight. Thus, traps were protected from even the largest amounts of dust if this technique of floor cleaning was not used; where it was used, the efficacy of the cap modification was diminished.

Research on movement and dispersal of *T. castaneum* adults is dominated by experiments with walking beetles in the laboratory orienting to traps, pheromones, or other stimuli [3,22,23,24], with very little work on beetle flight in nature. Recent research [25] studied flight initiation *T. castaneum* in a laboratory wind tunnel in response to various stimuli but not to aggregation pheromone in the field. Our data on capture of flying *T. castaneum* in pheromone-baited funnel traps outdoors (Figure 10) may be one of the earliest examples of this phenomenon [14]. Recent research in Australia studied the flight dispersal of adult *T. castaneum* to unbaited traps indoors and outdoors from heavily infested grain storages [26], and another study [27] used pheromone-baited Lindgren funnel traps, much like our experiment reported here, to trap *T. castaneum* up to 300 m away from grain storages. More work could be done with responses of *T. castaneum* to pheromone in flight to add to our knowledge of the dispersal and spread of this serious pest.

Pheromone-baited traps are important tools for detecting and monitoring stored-product pests as part of threshold-based decision-making in stored product integrated pest management [7]. Sticky traps, pitfall traps and flight traps can each have a habitat that is best for each to be used in detecting and monitoring adults of *T. castaneum*. A recent commercial version of the Dome trap described here, which is the pitfall trap for walking insects that is modified with a durable dust-cover, is used in research [22,28] and has seen extensive use by the grain, food, and pest control industries for integrated pest management [29].

## 5. Conclusions

A simple pitfall trap baited with an aggregation pheromone and a food-odor attractant is a sensitive tool for capturing red flour beetle adults, *Tribolium castaneum*. However, dusty environments can result in the pitfall trap operating poorly and not capturing beetles at an optimum level. This research showed that a dust cover can be applied to the top of a baited pitfall trap and significantly improve the capture of beetles in dusty environments of a flourmill. The dust cover was incorporated into the commercial pitfall trap product and now is known as the Dome trap, which is widely used for pest management of flour beetles and other stored product insects throughout the grain and food industries in many countries. Other traps were evaluated in this study for capturing *T. castaneum* and other flourmill insects with varying degrees of success. One experiment showed that in-flight adults of *T. castaneum* could be trapped in specialized flight traps baited with its synthetic aggregation pheromone.

## References

[1] Burkholder W.E., Ma M. (1985). Pheromones for monitoring and control of stored-product insects. Annu. Rev. Entomol..

[2] Phillips T.W., Cogan P.M., Fadamiro H.Y., Subramanyam B.H., Hagstrum D.W. (2000). Pheromones. Alternatives to Pesticides in Stored-Product IPM.

[3] Campbell J.F. (2012). Attraction of walking *Tribolium castaneum* adults to traps. J. Stored Prod. Res..

[4] Hussain A., Phillips T.W., AliNiazee M.T. Responses of *Tribolium castaneum* to different pheromone lure and traps in the laboratory. Proceedings of the 6th International Working Conference On Stored-Product Protection.

[5] Hussain A., Phillips T.W., Mayhew T.J., AliNiazee M.T. Pheromone biology and factors affecting its production in *Tribolium castaneum*. Proceedings of the 6th International Working Conference on Stored-Product Protection.

[6] Suzuki T. (1981). Identification of the aggregation pheromone of flour beetles *Tribolium castaneum* and *T. confusum* (Coleoptera: Tenebrionidae). Agric. Biol. Chem..

[7] Phillips T.W., Throne J.E. (2010). Biorational Approaches to Managing Stored-Product Insects. Ann. Rev. Entomol..

[8] Mullen M.A. (1992). Development of a pheromone trap for monitoring *Tribolium castaneum*. J. Stored Prod. Res..

[9] Chambers J. (1990). Overview on stored-product insect pheromones and food attractants. J. Kans. Entomol. Soc..

[10] Pinniger D.B. (1990). Food-baited traps; past, present, and future. J. Kans. Entomol. Soc..

[11] Barak A.V., Burkholder W.E., Faustini D.L. (1991). Factors affecting the design of traps for stored-product insects. J. Kans. Entomol. Soc..

[12] Mondal K.A.M.S.H., Port G.R. (1994). Pheromones of *Tribolium* spp. (Coleoptera: Tenebrionidae) and their potential in pest management. Agric. Zool. Rev..

[13] Tingle F.C., Mitchell E.R. (1975). Capture of *Spodiptera frugiperda* and *S. exigua* in pheromone traps. J. Econ. Entomol..

[14] Doud C.W. (1999). Monitoring the Red Flour Beetle, *Tribolium castaneum* (Herbst) (Coleoptera: Tenebrionidae) and Other Stored-Product Insects with Traps in Flour Mills. Master’s Thesis.

[15] Lingren B.S. (1983). A multiple funnel trap for scolytid beetles (Coleoptera). Can. Entomol..

[16] SAS Institute (1995). SAS for Windows Release 6.

[17] Stejskal V. (1995). The influence of food and shelter on the efficacy of a commercial sticky trap in *Tribolium castaneum* (Coleoptera: Tenebrionidae). J. Stored Prod. Res..

[18] Larson Z., Subramanyam B., Herman T. (2008). Stored-product insects associated with eight feed mills in the Midwestern United States. J. Econ. Entomol..

[19] Roesli R., Subramanyam B., Campbell J.F., Kemp K. (2003). Stored-product insects associated with a retail pet store chain in Kansas. J. Econ. Entomol..

[20] Raffa K.F., Phillips T.W., Salom S.M., Schowalter T., Filip G. (1993). Mechanisms and Strategies of Host Colonization by Bark Beetles. Interactions among Bark Beetles, Pathogens and Conifers in North American Forests.

[21] Landolt P.J., Phillips T.W. (1997). Host plant influences on sex pheromone behavior of phytophagous insects. Ann. Rev. Entomol..

[22] Gerken A.R., Scully E.D., Campbell J.F. (2018). Red flour beetle (Coleoptera: Tenebrionidae) response to volatile cues varies with strain and behavioral assay. Environ. Entomol..

[23] Dissanayaka D.M.S.K., Sammani A.M.P., Wijayaratne L.K.W. (2018). Aggregation pheromone 4.8-dimethyldecanal and kairomones affect the orientation of *Tribolium castaneum* (Herbst) (Coleoptera: Tenebrionidae) adults. J. Stored Prod. Res..

[24] Dissanayaka D.M.S.K., Sammani A.M.P., Wijayaratne L.K.W., Bamunuarachchige T.C., Morrison W.R. (2020). Distance and height of attraction by walking and flying beetles to traps with simultaneous use of the aggregation pheromones from *Tribolium castaneum* (Herbst) (Coleoptera: Tenebrionidae) and *Rhyzopertha dominica* (F.)(Coleoptera: Bostrychidae). J. Stored Prod. Res..

[25] Perez-Mendoza J., Campbell J.F., Throne J.E. (2014). Effect of abiotic factors on initiation of red flour beetle (Coleoptera: Tenebrionidae) flight. J. Econ. Entomol..

[26] Rafter M.A., Muralitharan V., Shandrasekaran S., Mohankumar S., Daglish G.J., Loganathan M., Walter G.H. (2019). Behavior in the presence of resource excess—Flight of *Tribolium castaneum* around heavily-infested grain storage facilities. J. Pest Sci..

[27] Gurdasania K., Rafter M.A., Daglish G.J., Walter G.H. (2019). The dispersal flight of *Tribolium castaneum*—A field test of laboratory generated predictions. J. Stored Prod. Res..

[28] McKay T., Bowombe-Toko M.P., Starkus L.A., Arthur F.H., Campbell J.F. (2019). Monitoring of *Tribolium castaneum* (Coleoptera: Tenebrionidae) in rice mills using pheromone-baited traps. J. Econ. Entomol..

[29] Toews M., Nansen C., Hagstrum D.W., Phillips T.W., Cuperus G. (2012). Trapping and Interpreting Captures of Stored Grain Insects. Stored Product Protection.

